# Parcellation of the Human Cerebral Cortex Based on Molecular Targets in the Serotonin System Quantified by Positron Emission Tomography *In vivo*

**DOI:** 10.1093/cercor/bhy249

**Published:** 2018-10-24

**Authors:** Gregory M James, Gregor Gryglewski, Thomas Vanicek, Neydher Berroterán-Infante, Cécile Philippe, Alexander Kautzky, Lukas Nics, Chrysoula Vraka, Godber M Godbersen, Jakob Unterholzner, Helen L Sigurdardottir, Marie Spies, René Seiger, Georg S Kranz, Andreas Hahn, Markus Mitterhauser, Wolfgang Wadsak, Andreas Bauer, Marcus Hacker, Siegfried Kasper, Rupert Lanzenberger

**Affiliations:** 1 Department of Psychiatry and Psychotherapy, Medical University of Vienna, Vienna, Austria; 2 Department of Biomedical Imaging and Image-guided Therapy, Division of Nuclear Medicine, Medical University of Vienna, Vienna, Austria; 3 Department of Rehabilitation Sciences, The Hong Kong Polytechnic University Hong Kong, China; 4 Ludwig Boltzmann Institute Applied Diagnostics, Vienna, Austria; 5 Center for Biomarker Research in Medicine (CBmed), Graz, Austria; 6 Institute of Neuroscience and Medicine (INM-2), Research Centre Jülich, Jülich, Germany

**Keywords:** cortex, cortical reconstruction, molecular imaging, parcellation, PET, serotonin

## Abstract

Parcellation of distinct areas in the cerebral cortex has a long history in neuroscience and is of great value for the study of brain function, specialization, and alterations in neuropsychiatric disorders. Analysis of cytoarchitectonical features has revealed their close association with molecular profiles based on protein density. This provides a rationale for the use of *in vivo* molecular imaging data for parcellation of the cortex with the advantage of whole-brain coverage. In the current work, parcellation was based on expression of key players of the serotonin neurotransmitter system. Positron emission tomography was carried out for the quantification of serotonin 1A (5-HT_1A_, *n* = 30) and 5-HT_2A_ receptors (*n* = 22), the serotonin-degrading enzyme monoamine oxidase A (MAO-A, *n* = 32) and the serotonin transporter (5-HTT, *n* = 24) in healthy participants. Cortical protein distribution maps were obtained using surface-based quantification. Based on k-means clustering, silhouette criterion and bootstrapping, five distinct clusters were identified as the optimal solution. The defined clusters proved of high explanatory value for the effects of psychotropic drugs acting on the serotonin system, such as antidepressants and psychedelics. Therefore, the proposed method constitutes a sensible approach towards integration of multimodal imaging data for research and development in neuropharmacology and psychiatry.

## Introduction

The classification of the cerebral cortex into discrete regions bears several advantages for the study of brain function. Ideally, the distinction of parcels is reproducible across individuals and laboratories, and accompanied by a nomenclature, such that specific scrutiny and scientific discourse is facilitated. Historically, Brodmann areas constitute the most prominent attempt in this direction and are based on Brodmann’s observations of cytoarchitectonical features of the cerebral cortex (Zilles and Amunts 2010). The advance of new technologies that allow for the measurement of different properties of human brain tissue has spurred attempts to improve parcellation. Especially in the field of brain imaging, a reliable reference system is indispensable (Amunts et al. 2014), as exemplified by the effort of Talairach–Tournoux to translate Brodmann areas into a standardized coordinate system (Talairach and Tournoux 1988). Based on macro-anatomical landmarks, this atlas was transformed into the widely used MNI template created from an average of a multitude of MRI scans, which resulted in a more accurate representation of the population’s neuroanatomy (Chau and McIntosh 2005). Atlases based on averages of multiple subjects do not entirely accommodate inter-individual variation, especially if their alignment is based on macro-anatomical landmarks, such as gyrification of the cortex (Desikan et al. 2006; Destrieux et al. 2010), as these do not necessarily coincide with cytoarchitectonical borders (Uylings et al. 2005). One promising solution has been proposed and validated using data from the human connectome project, which entails the acquisition of standardized structural and functional MR sequences for each subject and enables an individualized delineation of cortical areas using a machine-learning classifier (Glasser et al. 2016). Another invaluable approach is the construction of probabilistic cytoarchitectonical atlases derived from computational analysis of histological sections in a set of several brains (Eickhoff et al. 2005). The finding that inter-individual variations in functional MRI activation could be reduced by representing the cerebral cortex in a two-dimensional coordinate system has initiated the trend of surface-based analysis (Fischl, Sereno, and Dale 1999; Fischl, Sereno, Tootell, et al. 1999). The benefits of this methodology have led to its extensive application in processing of structural and functional MRI, as well as molecular imaging data (Greve et al. 2014) and the investigation of structural and functional connectivity.

In the domain of connectivity analysis, the identification of parcels using resting-state fMRI signal and graph theoretical (Power et al. 2011), independent component (Yeo et al. 2014), Markov random field (Schaefer et al. 2017), or spectral clustering analysis (Craddock et al. 2012) has yielded appealing results with regard to reproducibility and elucidation of information processing in the cerebral cortex. On the other hand, hierarchical clustering approaches to parcellation may parallel the development of specialization in the cerebral cortex, as demonstrated in structural connectivity data (Moreno-Dominguez et al. 2014). A MRI study in twins which delineated clusters based solely on the heritability of cortical surface area similarly revealed a hierarchical organization of cortical areas (Chen et al. 2013). Other invaluable approaches are derived from the analysis of functional connectivity networks in comparison to gene expression patterns (Ganglberger et al. 2017).

It could be argued that the field of parcellation of the cortex has undergone a gradual paradigm shift from dissecting maximally distinct areas towards the identification of the topology of coexisting sub-systems. Interestingly, borders between regions defined by cytoarchitectonical features were found to coincide to a great extent with those defined by receptor profiles using autoradiography (Zilles et al. 2002), suggesting that functional differentiation of the cerebral cortex may be paralleled by receptor compositions. Not only does this constitute complementary information and render multimodal support for the parcellation derived from cytoarchitectonics, but it provides a rationale for the utilization of molecular imaging data *in vivo* to study brain ontology. A linear relationship between protein densities measured using autoradiography and outcome measures derived from molecular imaging methods such as positron emission tomography (PET) has been demonstrated for several proteins (Savli et al. 2012; Beliveau et al. 2017). A critical advantage of molecular imaging is that acquisition of comprehensive whole-brain data is possible within a single scan, as opposed to effortful sectioning and staining of selected regions. We therefore propose clustering using PET imaging data of molecular targets as a complementary approach to parcellation of the cortical surface. We expect that clusters defined on the expression of molecular targets will be of special relevance for the analysis of pharmacological imaging data and therefore facilitate research and development in this field. The presented analysis is constrained to PET data on four proteins involved in serotonergic neurotransmission for which a representative sample is available. Inarguably the serotonergic system exerts a strong influence during brain development, modulation of a large set of brain functions and behavior, and is one of the most important targets of therapeutic intervention in neuropsychopharmacology. Extensive application of the proposed parcellation methods to different neurotransmitter systems can be expected with the emergence of data sharing initiatives in the molecular imaging field (Knudsen et al. 2016). The resulting clusters were compared to established cytoarchitectonic and functional brain parcellations and interpreted in light of pharmacological effects targeting the investigated binding proteins.

## Materials and Methods

### Participants and Imaging Procedures

The dataset used in this analysis comprises PET and structural 3T MR measurements of 108 healthy participants in total. Each subject underwent one PET scan for one target. For details on demographics and data acquisition see Table 1, or previous publications covering the data used here (Elmenhorst et al. 2012; Lanzenberger et al. 2007, 2012; Gryglewski et al. 2017; Vanicek et al. 2017). Subjects were recruited by advertisement and provided written informed consent according to the study procedures approved by the Ethics Committees at the Medical University of Vienna, Austria, and the Medical Faculty of the University of Düsseldorf, Germany. Participants underwent medical examinations to assure their physical and mental health. Subjects had no history of psychopharmacological drug treatment, substance abuse or diagnosis of psychiatric conditions according to the Diagnostic and Statistical Manual of Mental Disorders IV (DSM-IV). Scans were performed regardless of menstrual cycle of female participants.

**Table 1:** Data composition, demographics and acquisition parameters.

Protein	5-HT_1A_ receptor	5-HT_2A_ receptor	MAO-A	5-HTT
Radioligand	[*carbonyl*-^11^C]WAY-100635	[^18^F]altanserin	[^11^C]harmine	[^11^C]DASB
Outcome measure	BP_ND_	BP_P_	V_T_	BP_ND_
*N* (females)	30 (14)	22 (16)	32 (17)	24 (7)
Age (mean ± SD)	26.7 ± 6.8	40.7 ± 11.6	35.3 ± 10.5	29.4 ± 8.0
PET scanner (3D mode)	GE Advance PET scanner	Siemens ECAT Exact HR+ scanner	GE Advance PET scanner	GE Advance PET scanner
Tracer administration	Bolus	Bolus plus infusion (*K*_bol_ = 2.1 h)	Bolus	Bolus
Dynamic emission scan	90 min	60 min (starting 120 min after tracer bolus)	90 min	90 min
Blood samples	––	venous	arterial	––
MRI (T1-weighted structural images)	Bruker Medspec 3T, MPRAGE: voxel size 0.78 × 0.86 × 1.56 mm	Siemens Magnetom Trio 3T, MPRAGE: 1 × 1 × 1 mm	Siemens Tim Trio 3 T(*n* = 15): 0.88 × 0.47 × 0.47 mm; Siemens PRISMA 3 T(*n* = 17): 1.1 × 1 × 1 mm	Bruker Medspec 3 T (*n* = 3), MPRAGE: 0.78 × 0.86 × 1.56 mm; Philips Achieva 3 T(*n* = 11), FFE: 0.88 × 0.47 × 0.47 mm; Siemens mMR 3 T(*n* = 10), MPRAGE: 1.1 × 1 × 1 mm

PET and MRI data from a total of 108 healthy subjects was obtained for the current analysis.

PET imaging was carried out using optimized protocols with application of highly specific tracers for quantification of the 5-HT_1A_ receptor ([*carbonyl*-^11^C]WAY-100635), 5-HT_2A_ receptor ([^18^F]altanserin), MAO-A ([^11^C]harmine) and serotonin transporter 5-HTT ([^11^C]DASB). [*carbonyl-*^11^C]WAY-100635 has a tenfold lower affinity for dopamine D4 receptors than for 5-HT_1A_ receptors (Chemel et al. 2006; Martel et al. 2007). Based on the low expression of D4 receptor mRNA in the cortex, we do not expect a relevant influence on binding potentials in this region (Matsumoto et al. 1996). Changes in [^11^C]harmine binding up to 17% were observed in one study after altering endogenous monoamine concentrations pharmacologically (Sacher et al. 2012). The other tracers used were shown to be insensitive to changes in endogenous ligand concentrations, with changes in [^11^C]DASB binding being observed only after extreme pharmacological challenges performed in animal studies (Paterson et al. 2010).

### Image Preprocessing

PET images were motion corrected in SPM12 (Wellcome Trust Centre for Neuroimaging, London, UK; http://www.fil.ion.ucl.ac.uk/spm) by rigid realignment of every frame image to a median image, which consisted of a motion-free period, selected by visual inspection. Duration of frames were between 5 and 300 s, depending on the protocol and time point (frame length was increasing towards the end of the measurements to improve signal to noise ratio). The median PET images were co-registered to the individual MR images. The cortical surface was reconstructed using FreeSurfer 6.0 (Harvard Medical School, Boston, USA; http://www.surfer.nmr.mgh.harvard.edu) with individual T1-weighted MR images serving as input. In addition, every reconstruction was visually inspected and manually corrected for possible inaccuracies in segmentation of the cortical surface by setting control points or erasing falsely segmented areas and re-initiating reconstruction according to published procedures (McCarthy et al. 2015). Volume to surface projection was performed using the FreeSurfer function *mri_vol2surf*. The obtained registration parameters were subsequently applied to the motion-corrected dynamic PET images resulting in dynamic PET data in fsaverage surface space.

### Quantification of Molecular Targets

The dynamic PET data registered to the cortical surface served as input for the quantification of protein distributions, which was performed in MATLAB 8.2 (https://www.mathworks.com) using models specified below. PET data was smoothed prior to quantification using an 8-mm kernel in surface space. The resulting outcome measures (binding potential, BP_ND_, BP_P_, V_T_) are proportional to absolute density of available receptors (Innis et al. 2007).

#### 5-HT_1A_ Receptor

The cortical 5-HT_1A_ receptor binding was quantified by applying the multilinear reference tissue model (MRTM2) (Ichise et al. 2003). The insular cortex was used as high-uptake and the cerebellar white matter as reference region, due to the low 5-HT_1A_ concentration in this area (Parsey et al. 2005). The choice of quantification method for 5-HT_1A_ receptor binding potentials may affect results of group comparisons based on variability of receptors in the reference region (Parsey et al. 2010). This issue is not relevant to the current analysis, as a potentially different scaling of data is removed by normalization prior to clustering.

#### 5-HT_2A_ Receptor

5-HT_2A_ receptor binding potential (BP_P_) was quantified using radioactivity in brain and blood plasma at equilibrium during tracer infusion (Elmenhorst et al. 2012). Activity in the reference region (cerebellum) was subtracted from cortical activity and divided by plasma activity.

#### Serotonin Transporter

Modeling of 5-HTT binding was similar to that in previous publications (James et al. 2017). In brief, the MRTM2 was applied with thalamus serving as high-uptake and cerebellar gray matter as reference region to obtain the binding potential (BP_ND_; Parsey et al. 2006).

#### Monoamine Oxidase A

Arterial blood sampling was performed to obtain arterial input functions after correction for the presence of radioactive metabolites using high-performance liquid chromatography. Arterial input functions were derived from the kinetic modeling tool implemented in PMOD 3.509 (PMOD Technologies Ltd., Zurich, Switzerland; http://www.pmod.com). The arterial input functions and cortical activity were processed using Logan plots to obtain volume of distribution (*V*_T_) as the outcome measure for MAO-A binding (Logan et al. 1990; Ginovart et al. 2006).

### Clustering Method

For each protein, data was averaged across subjects resulting in population-based maps for the distribution of 5-HT_1A_ receptors, 5-HT_2A_ receptors, MAO-A and 5-HTT on the cortical surface. In order to avoid weighting in the parcellation procedure owing to the differently scaled outcome measures, binding data for each protein was *z*-scored, which resulted in values with a mean of 0 and a standard deviation of 1. The standardized mean maps for the four proteins were used as input for the *k*-means clustering algorithm. The initial starting values were chosen according to the *K*-means++ algorithm (Arthur and Vassilvitskii 2007). Clustering was performed with a squared Euclidean distance measure. The solution with the lowest within-cluster sums of point-to-centroid distances was selected from 50 replicates. To obtain a robust estimate of the optimal number of clusters, bootstrapping was performed using subsets of the PET data. One hundred subsets were generated by randomly selecting five subjects for each molecular target. For each subset, the optimal number of clusters was determined by the silhouette criterion using squared Euclidean distance (Rousseeuw 1987). Possible *K* were evaluated from 4 (corresponding to the number of proteins used for clustering) to a limit of 20, due to the tendency of the criterion values to decrease with an increasing number of clusters. The *K* indicated for the majority of subsets in bootstrapping was selected as the optimal solution for the entire dataset. While selection of the optimal number of clusters was performed on a combined dataset of both hemispheres for computational reasons, clustering of the entire dataset was performed both for each hemisphere separately and on a combined dataset of both hemispheres.

To evaluate the resulting clusters, we examined the overlap of parcellation based on serotonergic molecules with those of the population-average, landmark- and surface-based version of the Brodmann atlas (Van Essen 2005), as well as a cortical parcellation based on major functional connectivity networks (Yeo et al. 2011).

## Results

**Figure 1.**
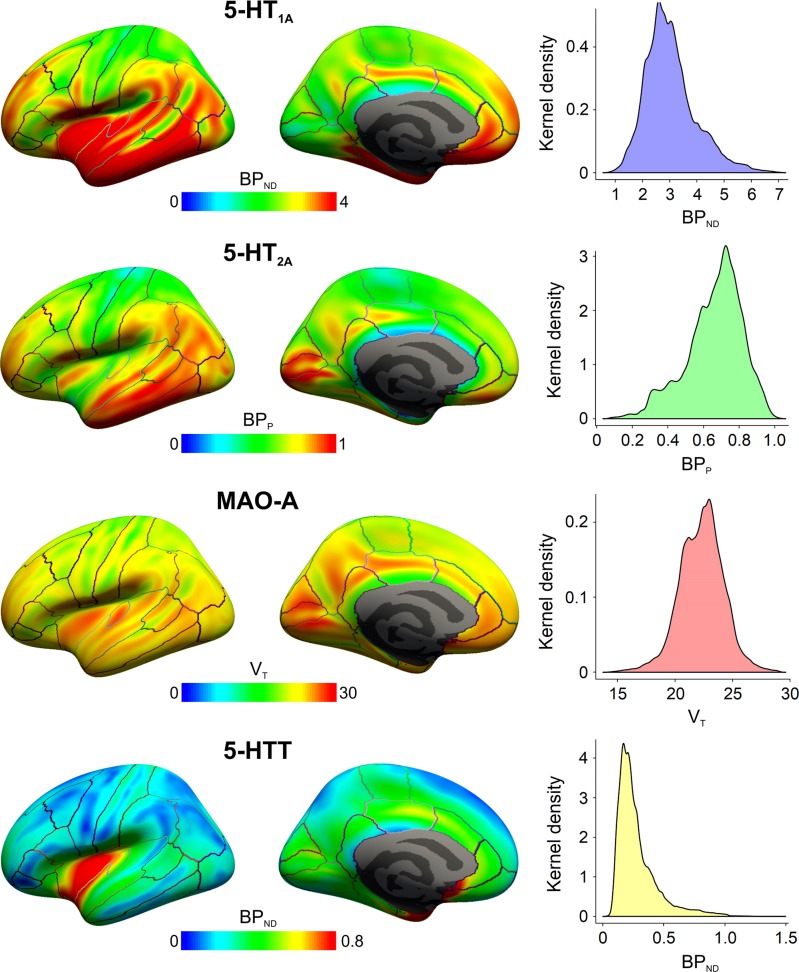
Population-based cortical protein binding maps. PET data on expression of four proteins centrally involved in modulatory serotonergic neurotransmission in the cerebral cortex is displayed. An inflated representation of the cortical surface is shown from a lateral (left) and mid-sagittal (middle) perspective. Kernel density plots illustrate the distribution of protein binding data in the average cortex (right). The kernel density is proportional to the number of data points at each value. Outcome measures (binding potentials (BP_ND_ or BP_P_) or volume of distribution (*V*_T_)) are proportional to absolute density of available protein. A total of 108 subjects were investigated, such that 22–32 individual scans were averaged to obtain each protein map.

**Figure 2.**
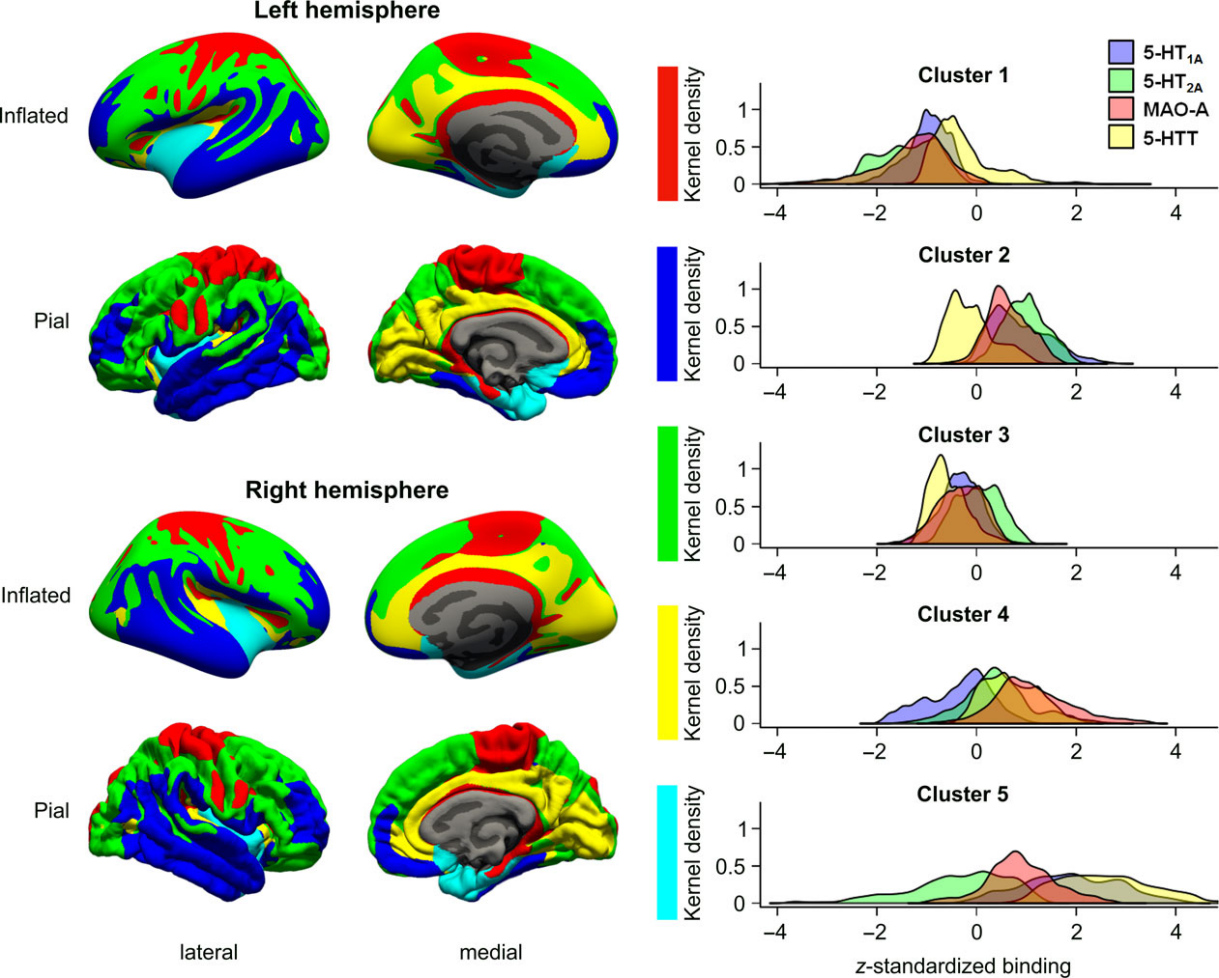
Topology and binding profiles of molecular clusters. Results of k-means clustering of cerebral cortex based on PET data of binding of 5-HT_1A_ and 5-HT_2A_ receptors, MAO-A and 5-HTT are summarized in this figure. The left and middle column display the allocation to one out of seven clusters for each coordinate on the cortical surface. Kernel density plots of standardized protein binding in each of the five clusters are shown in the right column. Corresponding absolute mean and standard deviations can be found in Table 2. Different perspectives on clustering results are displayed in Supplementary Figure 2.

**Figure 3.**
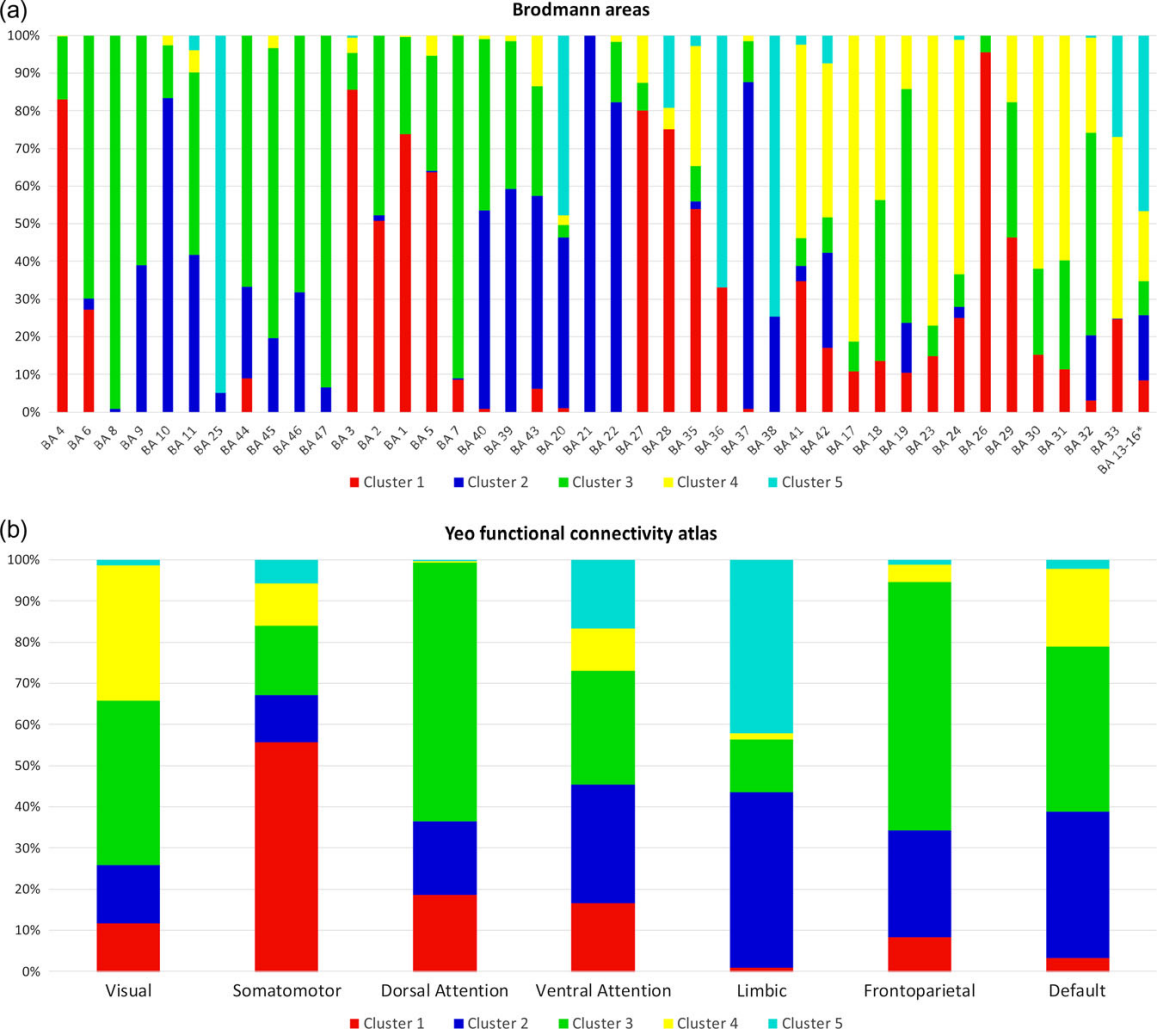
Overlap of molecular clusters with other cortical parcellation methods. The composition of region defined by different parcellation methods with respect to molecular clusters is displayed. For each region (*x*-axis), the fraction of the area allocated to each cluster is displayed as a bar summing to 100% on the *y*-axis. Regions were defined by (a) the cytoarchitectonical Brodmann areas (BA) and (b) the functional connectivity Yeo atlas. Complementary information on the distribution of each molecular cluster across regions defined by the other parcellation methods is shown in Supplementary table 1. *The Freesurfer version of the PALS Brodmann atlas misses the assignment of one area, which has a considerable overlap with the insular cortex. Thus, we summarized the unassigned region with Brodmann area 13-16 according to Brodmann and Garey (2006).

Cortical protein maps generated for 5-HT_1A_ receptors, 5-HT_2A_ receptors, MAO-A and 5-HTT were in good agreement with distributions reported in the literature (Figure 1, Table 2) (Varnas et al. 2004; Savli et al. 2012; Beliveau et al. 2017). The silhouette criterion indicated *K* = 5 as the optimal solution in 83 out of 100 subsets during bootstrapping. As results for combined and separate analysis of hemispheres were highly similar (Supplementary Figure 1), the subsequent paragraphs only refer to results of the combined analysis. The topology and molecular profiles of clusters are shown in Figure 2 and Table 2. The comparison with other cortical parcellation methods is shown in Figure 3 and Supplementary Table 1. Supplementary Figure 2 displays multiple perspectives of clustering results. Clustering results are available for download in NIfTI and FreeSurfer (.annot) file formats at http://www.meduniwien.ac.at/neuroimaging/parcellation.html. Properties of clusters are summarized in the following paragraphs. Numbering of the clusters is arbitrary.

**Table 2:** Protein binding profiles of molecular clusters.

	5-HT_1A_ receptor (BP_ND_)	5-HT_2A_ receptor (BP_P_)	MAO-A (V_T_)	5-HTT (BP_ND_)	Surface (%)
Cortical surface	3.04 ± 0.93	0.66 ± 0.16	22.30 ± 1.92	0.28 ± 0.17	100
Cluster 1	2.16 ± 0.48	0.43 ± 0.10	19.86 ± 1.40	0.23 ± 0.10	19.15
Cluster 2	3.86 ± 0.55	0.81 ± 0.07	23.52 ± 0.82	0.26 ± 0.08	23.86
Cluster 3	2.74 ± 0.40	0.67 ± 0.07	21.69 ± 0.87	0.19 ± 0.07	36.96
Cluster 4	2.70 ± 0.61	0.72 ± 0.11	24.48 ± 1.49	0.40 ± 0.10	12.71
Cluster 5	4.81 ± 0.90	0.61 ± 0.16	24.04 ± 1.32	0.71 ± 0.16	7.32

Mean and standard deviation of outcome measures for each cluster and protein are given. Corresponding kernel density plots of standardized binding data are shown in Figure 2.

### Cluster 1

Cluster 1 has the lowest binding of MAO-A, 5-HT_1A_, and 5-HT_2A_ receptors. The majority of its area is located on the pre- and postcentral gyri and the paracentral lobule and extends to the superior parietal gyrus. Another part can be found adjacent to the corpus callosum extending from the caudal anterior cingulate cortex to the retrosplenial isthmus cingulate cortex and parahippocampal gyrus (Brodmann areas (BA) 26-29 and 35). Almost two-thirds of this cluster correspond to the somatomotor network and comprise the majority of BA 1–5, 6, and 7.

### Cluster 2

Cluster 2 is characterized by the highest binding of excitatory 5-HT_2A_ receptors among all clusters and second highest expression of inhibitory 5-HT_1A_ receptors. The cluster spans 24% of the cortical surface. Two-thirds of the cluster are located in the superior, middle and inferior temporal gyri (BA 20-22 which contain Wernicke’s speech area), and extend to adjacent lateral occipital, fusiform (BA 37), angular, supramarginal, inferior parietal gyri (BA 39, 40). The remaining part of the cluster is located in the frontal cortex and comprises the majority of the rostral middle frontal gyrus (BA 9, 10, 46). Approximately 10% of the cluster are located in the medial and lateral orbitofrontal cortex (BA 11) and extend into the medial aspect of the superior frontal gyrus. Cluster 2 covers 30% of the default and 43% of the limbic networks.

### Cluster 3

Cluster 3 is the largest cluster and includes 37% of the cortical surface. Binding of 5-HTT is the lowest among all clusters, 5-HT_1A_ receptors and MAO-A are below average. One half of the cluster is located in the superior and inferior parietal cortex and extends into the precuneus (BA 7), lateral occipital (BA 19), supramarginal, and postcentral gyri. The remainder of the cluster can be found in the superior frontal gyrus (BA 8, 9) and covers the majority of the middle and inferior frontal (BA 45-47) gyri, as well as parts of the lateral orbitofrontal cortex and precentral gyrus (BA 6). Cluster 3 contributes 60% each to the dorsal attention and frontoparietal networks, and 40% each to the default mode and visual networks.

### Cluster 4

Cluster 4 has the highest binding of MAO-A and the second highest binding of 5-HTT. Binding of 5-HT_2A_ and 5-HT_1A_ receptors is slightly above and below average, respectively. The majority of Cluster 4 is located on the medial aspect of the cortex, spans from the rostral (BA 24, 33) to the posterior cingulate cortex (BA 23, 30, 31) and reaches into visual areas including the pre-transverse temporal gyri (BA 41, 42). Cluster 4 covers one-third of the visual and one-fifth of the default mode networks.

### Cluster 5

The highest binding of 5-HT_1A_ receptors and 5-HTT can be found in Cluster 5, and binding of MAO-A is ranked second. This cluster is the smallest and encompasses most of the insula (BA 13-16), temporal pole (BA 38) and entorhinal cortex and reaches out to the parahippocampal, fusiform (BA 36), and superior temporal gyri. Furthermore, Cluster 5 has an extension into the posterior parts of the orbitofrontal cortex and includes the subgenual area (BA 25). Cluster 5 contributes to 42% of the limbic network.

## Discussion

### Relevance of Molecular Clusters in the Serotonergic System

The current results represent a proof of principle for the parcellation of the cerebral cortex based on the topology of serotonergic molecular targets measured using PET. The convergence of clustering results in separate and combined analysis of left and right hemispheres provides internal validation for the method and indicates that clusters identified are not subject to a pronounced influence of lateralization. While *k* = 5 is a relatively low number of clusters compared to the number of regions identified by cytoarchitectonical features and the MR based methods discussed above, many of the allocations of cortical areas are consistent in synopsis with other methods. It must be kept in mind that the current clustering approach is merely based on four parameters, i.e., four different proteins involved in the same neurotransmitter system, such that differentiation of regions is achieved with respect to the distribution of these particular molecules. It is likely that the inclusion of PET data on other proteins will result in a finer parcellation of cortical regions. Nevertheless, the unique characteristics of each cluster provide a novel perspective on the nature of serotonergic neuromodulation in the cerebral cortex and hint at the potential utility of the proposed parcellation for the investigation of pharmacological effects in this system. In fact, it may be a sensible approach to tailor regions of interests for the analysis of pharmacological imaging data using clustering based on expression of molecular targets of the studied drugs (Gryglewski et al. 2018). If brain areas within clusters defined in this manner react uniformly to pharmacological intervention, a substantial increase in power can be expected. Multiple comparisons can be avoided in contrast to analyses based on atlases obtained from other parcellation methods which may suggest distinctions between brain areas that are not paralleled by a differential response to the effects of the studied drug.

In detail, Cluster 1 is almost exclusively covered by the somatomotor network located at the pre- and postcentral gyrus and it may be argued that the proposed parcellation method failed to identify the indisputable border between primary motor and somatosensory cortices. However, if we consider that Cluster 1 is characterized by the lowest expression of all proteins studied, we may hypothesize that the effect of serotonergic neuromodulation is relatively low in these areas after completion of brain development (D’Amato et al. 1987). This is in line with the lack of effects on brain activation of 5-HT_2A_ receptor agonists in this area (Kraehenmann et al. 2015). The adjacent Cluster 3 is characterized by the lowest binding of serotonin transporters, the expression of which has been found to be highly associated with extracellular serotonin levels (Dewar et al. 1991). Higher binding of serotonin receptors suggest that serotonergic neuromodulation may be effectuated in secondary and associative regions within Cluster 3, i.e. parts of the dorsal attention, frontoparietal, default mode and visual networks, at lower levels of serotonin or serotonin receptor binding drugs than in Cluster 1. Based on the low expression of 5-HTT, no pronounced effects of selective serotonin reuptake inhibitors (SSRIs) can be expected in clusters 1, 2, and 3. In contrast, Cluster 2 is characterized by relatively higher binding of MAO-A compared to 5-HTT and 30% and 43% of this cluster overlap with the default mode and limbic networks, respectively. These areas include the medial prefrontal cortex, temporoparietal junction, orbitofrontal cortex (Raichle and Snyder 2007; Sheline et al. 2009; Zhu et al. 2012), as well as the dorsolateral prefrontal cortex which is targeted with transcranial magnetic stimulation for the treatment of depression (Kolbinger et al. 1995; Pascual-Leone et al. 1996). This may underlie the response of patients to MAO inhibitors who have failed antidepressant treatment with SSRIs and justifies the independent role of the success of a trial with MAO inhibitors in staging of treatment resistant depression (Ruhe et al. 2012). The high expression of MAO-A in Cluster 4, 50% of which belongs to the visual network, may further underlie the increase of visual hallucinations with the addition of MAO inhibitors in the form of harmine (radiolabeled for the quantification of MAO-A in our study) and other harmala alkaloids to dimethyltryptamine in ayahuasca preparations, next to their influence on first-pass metabolism (McKenna et al. 1984; De Araujo et al. 2012). Given their high expression of 5-HT_2A_ receptors, Clusters 2 and 4 are implicated in the effects of drugs acting as agonists on these receptors, such as psilocybin and LSD, which is in line with psychedelic effects on visual and auditory perception and corresponding imaging studies on glucose metabolism, fMRI parameters and blood flow in these areas which were found to correlate with subjective drug effects (Vollenweider et al. 1997; Kraehenmann et al. 2015; Carhart-Harris et al. 2012, 2016). Lastly, with their high expression of 5-HTT Clusters 4 and 5 are most likely to be affected by the application of SSRIs which constitute the first line of treatment for depression and anxiety disorders. The extent of Cluster 4 in the cingulate and Cluster 5 in the parahippocampal and insular cortices includes regions that were historically assigned to the limbic system and are continuously under investigation for their role in emotion processing (Mayberg et al. 1999), empathy (Mitchell et al. 2013), and affective disorders (Montag et al. 2009; Horn et al. 2010; Klumpers et al. 2010). There is a remarkable overlap between these clusters and peak decreases in 5-HT_1A_ receptor binding following treatment with SSRIs (Spindelegger et al. 2009) or electroconvulsive therapy (Lanzenberger et al. 2013), and increases in gray matter volume following electroconvulsive therapy (Sartorius et al. 2016).

### Limitations

Next to the limitation that only four proteins were available for the current analysis, several other issues should be mentioned. Given the resolution of molecular imaging using PET, some areas may be affected by partial volume effects, that is blurring of signal at borders between adjacent regions. While partial volume effects are reduced by surface-based analysis (Greve et al. 2014), some regions may be still affected and result in the identification of clusters in areas of mixed signal. The delineation of Cluster 1 close to the corpus callosum is likely due to a partial volume effect rather than low binding in this area of the cingulate cortex. The localization of proteins on pre- or post-synaptic sites, excitatory or inhibitory neurons cannot be differentiated using PET, but is likely to vary across the cortex and affect the effects of pharmacological modulation. Another issue is the fact that all four proteins could not be quantified in the same subjects, to the end that covariances between these proteins are unknown and probabilistic information on the inter-individual variation of the extent of clusters could not be generated. However, the protein maps in this analysis are generated from an adequately sized sample of healthy subjects and should constitute a fair representation of average protein distribution in the healthy adult population. Nevertheless, the study of the influence of demographic variables on clustering results cannot be performed with this sample size. The use of a different scanner for 5-HT_2A_ receptor imaging may reduce the conformity of resulting PET data, which may have been accommodated by the uniform quantification pipeline, smoothing and standardization of the data. The focus of the current work was parcellation of the cortex. Clustering of cerebellum was not performed based on its negligible concentration of three out of four proteins studied, for the quantification of which it serves as reference region. Given the lack of a validated quantification pipeline which allows for integration of cortical surface-based outcome measures with volumetric subcortical regions, these would have to be processed separately. Furthermore, an optimized normalization pipeline for registration of PET data obtained from subcortical regions using different tracers is lacking. Lastly, at the present time we could only provide support for the utility of our method with reference to previous research in pharmacological imaging. Direct application of the defined clusters for analysis of imaging data is still pending.

### Concluding Remarks

Definition of brain regions using clustering of molecular imaging data results in a parcellation of the cerebral cortex which is of high explanatory value for the appraisal of the effects of psychotropic drugs. The application of parcels defined in this manner for the analysis of pharmacological imaging data *a priori* will increase sensitivity and power of these investigations using, e.g. functional magnetic resonance imaging, which is a pressing issue in the field. Therefore, the proposed method constitutes a highly promising approach towards integration of multimodal imaging data for research and development in neuropharmacology and psychiatry.

## Supplementary Material

Supplementary Data

## References

[bhy249C1] Amunts K, Hawrylycz MJ, Van Essen DC, Van Horn JD, Harel N, Poline JB, De Martino F, Bjaalie JG, Dehaene-Lambertz G, Dehaene S, et al 2014 Interoperable atlases of the human brain. Neuroimage. 99:525–532. 24936682 10.1016/j.neuroimage.2014.06.010

[bhy249C2] Arthur D, Vassilvitskii S 2007. k-means++: the advantages of careful seeding. In: Proceedings of the eighteenth annual ACM-SIAM symposium on Discrete algorithms. p. 1027–1025.

[bhy249C3] Beliveau V, Ganz M, Feng L, Ozenne B, Hojgaard L, Fisher PM, Svarer C, Greve DN, Knudsen GM 2017 A high-resolution *in vivo* atlas of the human brain’s serotonin system. J Neurosci. 37:120–128. 28053035 10.1523/JNEUROSCI.2830-16.2016 PMC5214625

[bhy249C4] Brodmann K, Garey LJ 2006 Brodmann’s Localisation in the Cerebral Cortex. New York (USA): Springer.

[bhy249C5] Carhart-Harris RL, Erritzoe D, Williams T, Stone JM, Reed LJ, Colasanti A, Tyacke RJ, Leech R, Malizia AL, Murphy K, et al 2012 Neural correlates of the psychedelic state as determined by fMRI studies with psilocybin. Proc Natl Acad Sci USA. 109:2138–2143. 22308440 10.1073/pnas.1119598109 PMC3277566

[bhy249C6] Carhart-Harris RL, Muthukumaraswamy S, Roseman L, Kaelen M, Droog W, Murphy K, Tagliazucchi E, Schenberg EE, Nest T, Orban C, et al 2016 Neural correlates of the LSD experience revealed by multimodal neuroimaging. Proc Natl Acad Sci USA. 113:4853–4858. 27071089 10.1073/pnas.1518377113 PMC4855588

[bhy249C7] Chau W, McIntosh AR 2005 The Talairach coordinate of a point in the MNI space: how to interpret it. Neuroimage. 25:408–416. 15784419 10.1016/j.neuroimage.2004.12.007

[bhy249C8] Chemel BR, Roth BL, Armbruster B, Watts VJ, Nichols DE 2006 WAY-100635 is a potent dopamine D4 receptor agonist. Psychopharmacology (Berl). 188:244–251. 16915381 10.1007/s00213-006-0490-4

[bhy249C71] Chen CH, Fiecas M, Gutiérrez ED, Panizzon MS, Eyler LT, Vuoksimaa E., Thompson WK, Fennema-Notestine C, Hagler DJ, Jernigan TL, et al 2013 Genetic topography of brain morphology. Proc Natl Acad Sci USA. 110:17089–17094. doi:10.1073/pnas.1308091110. 24082094 10.1073/pnas.1308091110 PMC3801007

[bhy249C9] Craddock RC, James GA, Holtzheimer PE, Hu XP, Mayberg HS 2012 A whole brain fMRI atlas generated via spatially constrained spectral clustering. Hum Brain Mapp. 33:1914–1928. 21769991 10.1002/hbm.21333 PMC3838923

[bhy249C10] De Araujo DB, Ribeiro S, Cecchi GA, Carvalho FM, Sanchez TA, Pinto JP, de Martinis BS, Crippa JA, Hallak JEC, Santos AC 2012 Seeing with the eyes shut: neural basis of enhanced imagery following ayahuasca ingestion. Hum Brain Mapp. 33:2550–2560. 21922603 10.1002/hbm.21381 PMC6870240

[bhy249C11] Desikan RS, Ségonne F, Fischl B, Quinn BT, Dickerson BC, Blacker D, Buckner RL, Dale AM, Maguire RP, Hyman BT, et al 2006 An automated labeling system for subdividing the human cerebral cortex on MRI scans into gyral based regions of interest. Neuroimage. 31:968–980. 16530430 10.1016/j.neuroimage.2006.01.021

[bhy249C12] Destrieux C, Fischl B, Dale A, Halgren E 2010 Automatic parcellation of human cortical gyri and sulci using standard anatomical nomenclature. Neuroimage. 53:1–15. 20547229 10.1016/j.neuroimage.2010.06.010 PMC2937159

[bhy249C13] Dewar KM, Reader TA, Grondin L, Descarries L 1991 [3H]Paroxetine binding and serotonin content of rat and rabbit cortical areas, hippocampus, neostriatum, ventral mesencephalic tegmentum, and midbrain raphe nuclei region. Synapse. 9:14–26. 1724575 10.1002/syn.890090104

[bhy249C14] D’Amato RJ, Blue ME, Largent BL, Lynch DR, Ledbetter DJ, Molliver ME, Snyder SH 1987 Ontogeny of the serotonergic projection to rat neocortex: transient expression of a dense innervation to primary sensory areas. Proc Natl Acad Sci USA. 84:4322–4326. 3473503 10.1073/pnas.84.12.4322 PMC305077

[bhy249C15] Eickhoff SB, Stephan KE, Mohlberg H, Grefkes C, Fink GR, Amunts K, Zilles K 2005 A new SPM toolbox for combining probabilistic cytoarchitectonic maps and functional imaging data. Neuroimage. 25:1325–1335. 15850749 10.1016/j.neuroimage.2004.12.034

[bhy249C16] Elmenhorst D, Kroll T, Matusch A, Bauer A 2012 Sleep deprivation increases cerebral serotonin 2a receptor binding in humans. Sleep. 35:1615–1623. 23204604 10.5665/sleep.2230 PMC3490354

[bhy249C17] Fischl B, Sereno MI, Dale AM 1999 Cortical surface-based analysis. Neuroimage. 9:195–207. 9931269 10.1006/nimg.1998.0396

[bhy249C18] Fischl B, Sereno MI, Tootell RBH, Dale AM 1999 High-resolution inter-subject averaging and a surface-based coordinate system. Hum Brain Mapp. 8:272–284. 10619420 10.1002/(SICI)1097-0193(1999)8:4<272::AID-HBM10>3.0.CO;2-4 PMC6873338

[bhy249C19] Ganglberger F, Kaczanowska J, Penninger JM, Hess A, Bühler K, Haubensak W 2017 Predicting functional neuroanatomical maps from fusing brain networks with genetic information. Neuroimage. 170:113–120. 28877513 10.1016/j.neuroimage.2017.08.070

[bhy249C20] Ginovart N, Meyer JH, Boovariwala A, Hussey D, Rabiner EA, Houle S, Wilson AA 2006 Positron emission tomography quantification of [11C]-harmine binding to monoamine oxidase-A in the human brain. J Cereb Blood Flow Metab. 26:330–344. 16079787 10.1038/sj.jcbfm.9600197

[bhy249C21] Glasser MF, Coalson TS, Robinson EC, Hacker CD, Harwell J, Yacoub E, Ugurbil K, Andersson J, Beckmann CF, Jenkinson M, et al 2016 A multi-modal parcellation of human cerebral cortex. Nature. 536:171–178. 27437579 10.1038/nature18933 PMC4990127

[bhy249C22] Greve DN, Svarer C, Fisher PM, Feng L, Hansen AE, Baare W, Rosen B, Fischl B, Knudsen GM 2014 Cortical surface-based analysis reduces bias and variance in kinetic modeling of brain PET data. Neuroimage. 92:225–236. 24361666 10.1016/j.neuroimage.2013.12.021 PMC4008670

[bhy249C23] Gryglewski G, Rischka L, Philippe C, Hahn A, James GM, Klebermass E, Hienert M, Silberbauer L, Vanicek T, Kautzky A, et al 2017 Simple and rapid quantification of serotonin transporter binding using [11C]DASB bolus plus constant infusion. Neuroimage. 149:23–32. 28119137 10.1016/j.neuroimage.2017.01.050

[bhy249C70] Gryglewski G, Seiger R, James GM, Godbersen GM, Komorowski A, Unterholzner J, Michenthaler P, Hahn A, Wadsak W, Mitterhauser M, et al 2018 Spatial analysis and high resolution mapping of the human whole-brain transcriptome for integrative analysis in neuroimaging. 176:259–267. 10.1016/j.neuroimage.2018.04.068 29723639

[bhy249C24] Horn DI, Yu C, Steiner J, Buchmann J, Kaufmann J, Osoba A, Eckert U, Zierhut KC, Schiltz K, He H, et al 2010 Glutamatergic and resting-state functional connectivity correlates of severity in major depression - the role of pregenual anterior cingulate cortex and anterior insula. Front Syst Neurosci. 4:1–10. 20700385 10.3389/fnsys.2010.00033 PMC2914530

[bhy249C25] Ichise M, Liow JS, Lu JQ, Takano A, Model K, Toyama H, Suhara T, Suzuki K, Innis RB, Carson RE 2003 Linearized reference tissue parametric imaging methods: application to [11C]DASB positron emission tomography studies of the serotonin transporter in human brain. J Cereb Blood Flow Metab. 23:1096–1112. 12973026 10.1097/01.WCB.0000085441.37552.CA

[bhy249C26] Innis RB, Cunningham VJ, Delforge J, Fujita M, Gjedde A, Gunn RN, Holden J, Houle S, Huang SC, Ichise M, et al 2007 Consensus nomenclature for in vivo imaging of reversibly binding radioligands. J Cereb Blood Flow Metab. 27:1533–1539. 17519979 10.1038/sj.jcbfm.9600493

[bhy249C27] James GM, Baldinger-Melich P, Philippe C, Kranz GS, Vanicek T, Hahn A, Gryglewski G, Hienert M, Spies M, Traub-Weidinger T, et al 2017 Effects of selective serotonin reuptake inhibitors on interregional relation of serotonin transporter availability in major depression. Front Hum Neurosci. 11:1–10. 28220069 10.3389/fnhum.2017.00048 PMC5292566

[bhy249C28] Klumpers UMH, Veltman DJ, Drent ML, Boellaard R, Comans EFI, Meynen G, Lammertsma A a, Hoogendijk WJG 2010 Reduced parahippocampal and lateral temporal GABAA-[11C]flumazenil binding in major depression: preliminary results. Eur J Nucl Med Mol Imaging. 37:565–574. 19890631 10.1007/s00259-009-1292-9

[bhy249C29] Knudsen GM, Jensen PS, Erritzoe D, Baare WF, Ettrup A, Fisher PM, Gillings N, Hansen HD, Hansen LK, Hasselbalch SG, et al 2016 The Center for Integrated Molecular Brain Imaging (Cimbi) database. Neuroimage. 124:1213–1219. 25891375 10.1016/j.neuroimage.2015.04.025

[bhy249C30] Kolbinger HM, Höflich G, Hufnagel A, Müller H-J, Kasper S 1995 Transcranial magnetic stimulation (TMS) in the treatment of major depression—a pilot study. Hum Psychopharmacol Clin Exp. 10:305–310.

[bhy249C31] Kraehenmann R, Preller KH, Scheidegger M, Pokorny T, Bosch OG, Seifritz E, Vollenweider FX 2015 Psilocybin-induced decrease in amygdala reactivity correlates with enhanced positive mood in healthy volunteers. Biol Psychiatry. 78:572–581. 24882567 10.1016/j.biopsych.2014.04.010

[bhy249C32] Lanzenberger R, Baldinger P, Hahn A, Ungersboeck J, Mitterhauser M, Winkler D, Micskei Z, Stein P, Karanikas G, Wadsak W, et al 2013 Global decrease of serotonin-1A receptor binding after electroconvulsive therapy in major depression measured by PET. Mol Psychiatry. 18(1):93–100. 22751491 10.1038/mp.2012.93 PMC3526726

[bhy249C33] Lanzenberger R, Kranz GS, Haeusler D, Akimova E, Savli M, Hahn A, Mitterhauser M, Spindelegger C, Philippe C, Fink M, et al 2012 Prediction of SSRI treatment response in major depression based on serotonin transporter interplay between median raphe nucleus and projection areas. Neuroimage. 63:874–881. 22828162 10.1016/j.neuroimage.2012.07.023

[bhy249C34] Lanzenberger R, Mitterhauser M, Spindelegger C, Wadsak W, Klein N, Mien LK, Holik A, Attarbaschi T, Mossaheb N, Sacher J, et al 2007 Reduced serotonin-1A receptor binding in social anxiety disorder. Biol Psychiatry. 61:1081–1089. 16979141 10.1016/j.biopsych.2006.05.022

[bhy249C35] Logan J, Fowler JS, Volkow ND, Wolf AP, Dewey SL, Schlyer DJ, MacGregor RR, Hitzemann R, Bendriem B, Gatley SJ, et al 1990 Graphical analysis of reversible radioligand binding from time-activity measurements applied to [N-11C-methyl]-(-)-cocaine PET studies in human subjects. J Cereb Blood Flow Metab. 10:740–747. 2384545 10.1038/jcbfm.1990.127

[bhy249C36] Martel JC, Leduc N, Ormière AM, Faucillon V, Danty N, Culie C, Cussac D, Newman-Tancredi A 2007 WAY-100635 has high selectivity for serotonin 5-HT1A versus dopamine D4 receptors. Eur J Pharmacol. 574:15–19. 17854799 10.1016/j.ejphar.2007.07.015

[bhy249C37] Matsumoto M, Hidaka K, Tada S, Tasaki Y, Yamaguchi T 1996 Low levels of mRNA for dopamine D4 receptor in human cerebral cortex and striatum. J Neurochem. 66:915–919. 8769849 10.1046/j.1471-4159.1996.66030915.x

[bhy249C38] Mayberg HS, Liotti M, Brannan SK, McGinnis S, Mahurin RK, Jerabek PA, Silva JA, Tekell JL, Martin CC, Lancaster JL, et al 1999 Reciprocal limbic-cortical function and negative mood: converging PET findings in depression and normal sadness. Am J Psychiatry. 156:675–682. 10327898 10.1176/ajp.156.5.675

[bhy249C39] McCarthy CS, Ramprashad A, Thompson C, Botti JA, Coman IL, Kates WR 2015 A comparison of FreeSurfer-generated data with and without manual intervention. Front Neurosci. 9:1–18. 26539075 10.3389/fnins.2015.00379 PMC4612506

[bhy249C40] McKenna DJ, Towers GHN, Abbott F 1984 Monoamine oxidase inhibitors in South American hallucinogenic plants: Tryptamine and β-carboline constituents of Ayahuasca. J Ethnopharmacol. 10:195–223. 6587171 10.1016/0378-8741(84)90003-5

[bhy249C41] Mitchell JP, Heatherton TF, Macrae CN 2013 Distinct neural systems subserve person and object knowledge In: Social Neuroscience: Key Readings. New York (USA): Psychology Pres p. 53–62. 10.1073/pnas.232395699 PMC137574 12417766

[bhy249C42] Montag C, Weber B, Fliessbach K, Elger C, Reuter M 2009 The BDNF Val66Met polymorphism impacts parahippocampal and amygdala volume in healthy humans: Incremental support for a genetic risk factor for depression. Psychol Med. 39:1831–1839. 19335934 10.1017/S0033291709005509

[bhy249C43] Moreno-Dominguez D, Anwander A, Knösche TR 2014 A hierarchical method for whole-brain connectivity-based parcellation. Hum Brain Mapp. 35:5000–5025. 24740833 10.1002/hbm.22528 PMC6869099

[bhy249C44] Parsey RV, Arango V, Olvet DM, Oquendo M a, Van Heertum RL, John Mann J 2005 Regional heterogeneity of 5-HT1A receptors in human cerebellum as assessed by positron emission tomography. J Cereb Blood Flow Metab. 25:785–793. 15716853 10.1038/sj.jcbfm.9600072

[bhy249C45] Parsey RV, Kent JM, Oquendo MA, Richards MC, Pratap M, Cooper TB, Arango V, Mann JJ 2006 Acute occupancy of brain serotonin transporter by sertraline as measured by [11C]DASB and positron emission tomography. Biol Psychiatry. 59:821–828. 16213473 10.1016/j.biopsych.2005.08.010

[bhy249C46] Parsey RV, Ogden RT, Miller JM, Tin A, Hesselgrave N, Goldstein E, Mikhno A, Milak M, Zanderigo F, Sullivan GM, et al 2010 Higher serotonin 1A binding in a second major depression cohort: modeling and reference region considerations. Biol Psychiatry. 68:170–178. 20497898 10.1016/j.biopsych.2010.03.023 PMC2900398

[bhy249C47] Pascual-Leone A, Rubio B, Pallardó F, Catalá MD 1996 Rapid-rate transcranial magnetic stimulation of left dorsolateral prefrontal cortex in drug-resistant depression. Lancet. 348:233–237. 8684201 10.1016/s0140-6736(96)01219-6

[bhy249C48] Paterson LM, Tyacke RJ, Nutt DJ, Knudsen GM 2010 Measuring endogenous 5-HT release by emission tomography: promises and pitfalls. J Cereb Blood Flow Metab. 30:1682–1706. 20664611 10.1038/jcbfm.2010.104 PMC3023404

[bhy249C49] Power JD, Cohen AL, Nelson SM, Wig GS, Barnes KA, Church JA, Vogel AC, Laumann TO, Miezin FM, Schlaggar BL, et al 2011 Functional network organization of the human brain. Neuron. 72:665–678. 22099467 10.1016/j.neuron.2011.09.006 PMC3222858

[bhy249C50] Raichle ME, Snyder AZ 2007 A default mode of brain function: a brief history of an evolving idea. Neuroimage. 37:1083–1090. 17719799 10.1016/j.neuroimage.2007.02.041

[bhy249C51] Rousseeuw PJ 1987 Silhouettes: a graphical aid to the interpretation and validation of cluster analysis. J Comput Appl Math. 20:53–65.

[bhy249C52] Ruhe HG, van Rooijen G, Spijker J, Peeters FP, Schene AH 2012 Staging methods for treatment resistant depression. A systematic review. J Affect Disord. 137:35–45. 21435727 10.1016/j.jad.2011.02.020

[bhy249C53] Sacher J, Rabiner EA, Clark M, Rusjan P, Soliman A, Boskovic R, Kish SJ, Wilson AA, Houle S, Meyer JH 2012 Dynamic, adaptive changes in MAO-A binding after alterations in substrate availability: an in vivo 11C-harmine positron emission tomography study. J Cereb Blood Flow Metab. 32:443–446. 22186668 10.1038/jcbfm.2011.184 PMC3293124

[bhy249C54] Sartorius A, Demirakca T, Böhringer A, Clemm von Hohenberg C, Aksay SS, Bumb JM, Kranaster L, Ende G 2016 Electroconvulsive therapy increases temporal gray matter volume and cortical thickness. Eur Neuropsychopharmacol. 26:506–517. 26792445 10.1016/j.euroneuro.2015.12.036

[bhy249C55] Savli M, Bauer A, Mitterhauser M, Ding YS, Hahn A, Kroll T, Neumeister A, Haeusler D, Ungersboeck J, Henry S, et al 2012 Normative database of the serotonergic system in healthy subjects using multi-tracer PET. Neuroimage. 63:447–459. 22789740 10.1016/j.neuroimage.2012.07.001

[bhy249C56] Schaefer A, Kong R, Gordon EM, Laumann TO, Zuo X-N, Holmes AJ, Eickhoff SB, Yeo BTT 2017 Local-global parcellation of the human cerebral cortex from intrinsic functional connectivity MRI. Cereb Cortex. 28:1–20. 10.1093/cercor/bhx179 PMC6095216 28981612

[bhy249C57] Sheline YI, Barch DM, Price JL, Rundle MM, Vaishnavi SN, Snyder AZ, Mintun MA, Wang S, Coalson RS, Raichle ME 2009 The default mode network and self-referential processes in depression. Proc Natl Acad Sci USA. 106:1942–1947. 19171889 10.1073/pnas.0812686106 PMC2631078

[bhy249C58] Spindelegger C, Lanzenberger R, Wadsak W, Mien LK, Stein P, Mitterhauser M, Moser U, Holik A, Pezawas L, Kletter K, et al 2009 Influence of escitalopram treatment on 5-HT 1A receptor binding in limbic regions in patients with anxiety disorders. Mol Psychiatry. 14:1040–1050. 18362913 10.1038/mp.2008.35

[bhy249C59] Talairach J, Tournoux P 1988 Co-Planar Stereotaxis Atlas of the Human Brain. Stuttgart (Germany): G. Thieme.

[bhy249C60] Uylings HBM, Rajkowska G, Sanz-Arigita E, Amunts K, Zilles K 2005 Consequences of large interindividual variability for human brain atlases: converging macroscopical imaging and microscopical neuroanatomy. Anat Embryol (Berl). 210:423–431. 16180019 10.1007/s00429-005-0042-4

[bhy249C61] Van Essen DC 2005 A Population-Average, Landmark- and Surface-based (PALS) atlas of human cerebral cortex. Neuroimage. 28:635–662. 16172003 10.1016/j.neuroimage.2005.06.058

[bhy249C62] Vanicek T, Kutzelnigg A, Philippe C, Sigurdardottir HL, James GM, Hahn A, Kranz GS, Hoflich A, Kautzky A, Traub-Weidinger T, et al 2017 Altered interregional molecular associations of the serotonin transporter in attention deficit/hyperactivity disorder assessed with PET. Hum Brain Mapp. 38:792–802. 27770470 10.1002/hbm.23418 PMC6867013

[bhy249C63] Varnas K, Halldin C, Hall H 2004 Autoradiographic distribution of serotonin transporters and receptor subtypes in human brain. Hum Brain Mapp. 22:246–260. 15195291 10.1002/hbm.20035 PMC6872082

[bhy249C64] Vollenweider FX, Leenders KL, Scharfetter C, Maguire P, Stadelmann O, Angst J 1997 Positron emission tomography and fluorodeoxyglucose studies of metabolic hyperfrontality and psychopathology in the psilocybin model of psychosis. Neuropsychopharmacology. 16:357–372. 9109107 10.1016/S0893-133X(96)00246-1

[bhy249C65] Yeo BTT, Krienen FM, Chee MWL, Buckner RL 2014 Estimates of segregation and overlap of functional connectivity networks in the human cerebral cortex. Neuroimage. 88:212–227. 24185018 10.1016/j.neuroimage.2013.10.046 PMC4007373

[bhy249C66] Yeo BTT, Krienen FM, Sepulcre J, Sabuncu MR, Lashkari D, Hollinshead M, Roffman JL, Smoller JW, Zollei L, Polimeni JR, et al 2011 The organization of the human cerebral cortex estimated by intrinsic functional connectivity. J Neurophysiol. 106:1125–1165. 21653723 10.1152/jn.00338.2011 PMC3174820

[bhy249C67] Zhu X, Wang X, Xiao J, Liao J, Zhong M, Wang W, Yao S 2012 Evidence of a dissociation pattern in resting-state default mode network connectivity in first-episode, treatment-naive major depression patients. Biol Psychiatry. 71:611–617. 22177602 10.1016/j.biopsych.2011.10.035

[bhy249C68] Zilles K, Amunts K 2010 Centenary of Brodmann’s map conception and fate. Nat Rev Neurosci. 11:139–145. 20046193 10.1038/nrn2776

[bhy249C69] Zilles K, Palomero-Gallagher N, Grefkes C, Scheperjans F, Boy C, Amunts K, Schleicher A 2002 Architectonics of the human cerebral cortex and transmitter receptor fingerprints: reconciling functional neuroanatomy and neurochemistry. Eur Neuropsychopharmacol. 12:587–599. 12468022 10.1016/s0924-977x(02)00108-6

