# Supplementary material for: Parcellation of the Human Cerebral Cortex Based on Molecular Targets in the Serotonin System Quantified by Positron Emission Tomography In vivo
Source: Cereb Cortex. 2018 Oct 24;29(1):372–82. doi: 10.1093/cercor/bhy249 (PMC6294402; doi:10.1093/cercor/bhy249)
Supplement: Supplementary Data [file bhy249_supplementary_materials.zip › bhy249_James_Gryglewski_2018_Supplementary_Table_1.docx]

Supplementary Table 1a. Cluster distribution across regions from the cytoarchitectonical Brodmann areas (BA)

| **Cluster** | **Cluster 1** | **Cluster 2** | **Cluster 3** | **Cluster 4** | **Cluster 5** |
| --- | --- | --- | --- | --- | --- |
| **BA 4** | 27.9 |  | 2.8 | 0.1 |  |
| **BA 6** | 8.3 | 0.7 | 10.5 |  |  |
| **BA 8** |  | 0.1 | 6.5 |  |  |
| **BA 9** |  | 4.9 | 4.9 |  |  |
| **BA 10** |  | 8.2 | 0.9 | 0.5 |  |
| **BA 11** |  | 6.2 | 4.7 | 1.6 | 1.9 |
| **BA 25** |  | 0.1 |  |  | 5.1 |
| **BA 44** | 0.7 | 1.5 | 2.6 |  |  |
| **BA 45** |  | 0.6 | 1.5 | 0.2 |  |
| **BA 46** |  | 2.5 | 3.5 |  |  |
| **BA 47** |  | 0.1 | 1.2 |  |  |
| **BA 3** | 8.6 |  | 0.5 | 0.6 | 0.1 |
| **BA 2** | 10.6 | 0.2 | 4.9 |  |  |
| **BA 1** | 8.5 |  | 1.5 | 0.1 |  |
| **BA 5** | 6.5 |  | 1.5 | 0.8 |  |
| **BA 7** | 3.1 | 0.1 | 16.2 |  |  |
| **BA 40** | 0.2 | 7.6 | 4.2 | 0.2 |  |
| **BA 39** |  | 7.2 | 3.1 | 0.3 |  |
| **BA 43** | 0.4 | 2.4 | 0.9 | 1.2 |  |
| **BA 20** | 0.2 | 5.8 | 0.3 | 0.6 | 20.3 |
| **BA 21** |  | 6.8 |  |  |  |
| **BA 22** |  | 18.8 | 2.4 | 0.7 |  |
| **BA 27** | 0.7 |  |  | 0.2 |  |
| **BA 28** | 0.3 |  |  |  | 0.2 |
| **BA 35** | 0.7 |  | 0.1 | 0.6 | 0.1 |
| **BA 36** | 0.8 |  |  |  | 4.3 |
| **BA 37** | 0.2 | 11.7 | 0.9 | 0.4 |  |
| **BA 38** |  | 1.9 |  |  | 17.9 |
| **BA 41** | 1.7 | 0.2 | 0.2 | 3.7 | 0.3 |
| **BA 42** | 1.3 | 1.5 | 0.4 | 4.6 | 1.4 |
| **BA 17** | 1.4 |  | 0.5 | 14.6 |  |
| **BA 18** | 3.1 |  | 4.8 | 14.4 |  |
| **BA 19** | 3.8 | 3.7 | 11.3 | 7.5 |  |
| **BA 23** | 1.0 |  | 0.3 | 7.1 |  |
| **BA 24** | 3.7 | 0.3 | 0.6 | 13.4 | 0.4 |
| **BA 26** | 0.4 |  |  |  |  |
| **BA 29** | 0.3 |  | 0.1 | 0.2 |  |
| **BA 30** | 0.3 |  | 0.2 | 1.8 |  |
| **BA 31** | 1.3 |  | 1.6 | 9.8 |  |
| **BA 32** | 0.3 | 1.3 | 2.6 | 3.6 | 0.1 |
| **BA 33** | 0.2 |  |  | 0.5 | 0.5 |
| **BA 13-16*** | 3.4 | 5.4 | 1.8 | 10.9 | 47.5 |

Supplementary Table 1b. Cluster distribution across regions from the functional Yeo atlas

| **Cluster** | **Cluster 1** | **Cluster 2** | **Cluster 3** | **Cluster 4** | **Cluster 5** |
| --- | --- | --- | --- | --- | --- |
| **Visual** | 9.2 | 8.7 | 15.9 | 38.1 | 2.6 |
| **Somatomotor** | 58.8 | 9.6 | 9.1 | 16.1 | 15.8 |
| **Dorsal Attention** | 11.5 | 8.8 | 19.9 | 0.3 | 0.5 |
| **Ventral Attention** | 10.8 | 14.8 | 9.2 | 9.9 | 28.4 |
| **Limbic** | 0.4 | 13.6 | 2.6 | 0.9 | 44.7 |
| **Frontoparietal** | 5.8 | 14.1 | 21.2 | 4.3 | 1.9 |
| **Default** | 3.6 | 30.5 | 22.1 | 30.4 | 6.1 |

**Supplementary Table 1: Overlap of molecular clusters with other parcellations.** Distribution of each molecular cluster across regions delineated by other parcellation methods. Each column describes the percentage of area of each cluster falling within regions defined by (a) the cytoarchitectonical Brodmann areas (BA) and (b) the functional connectivity Yeo atlas. Values <0.05 are not shown.
