# Supplementary figures and images for: Parcellation of the Human Cerebral Cortex Based on Molecular Targets in the Serotonin System Quantified by Positron Emission Tomography In vivo
Source: Cereb Cortex. 2018 Oct 24;29(1):372–82. doi: 10.1093/cercor/bhy249 (PMC6294402; doi:10.1093/cercor/bhy249)

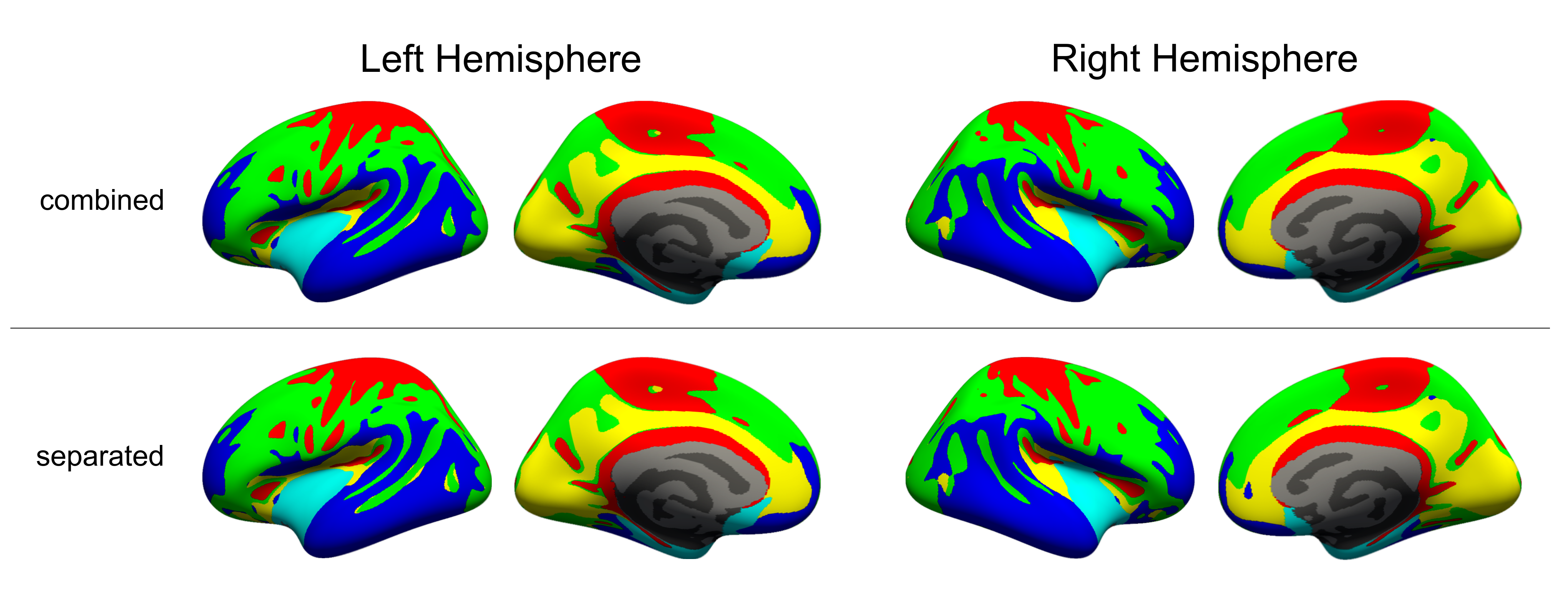

Supplement: Supplementary Data [file bhy249_supplementary_materials.zip › bhy249_Figure_1s.tif]

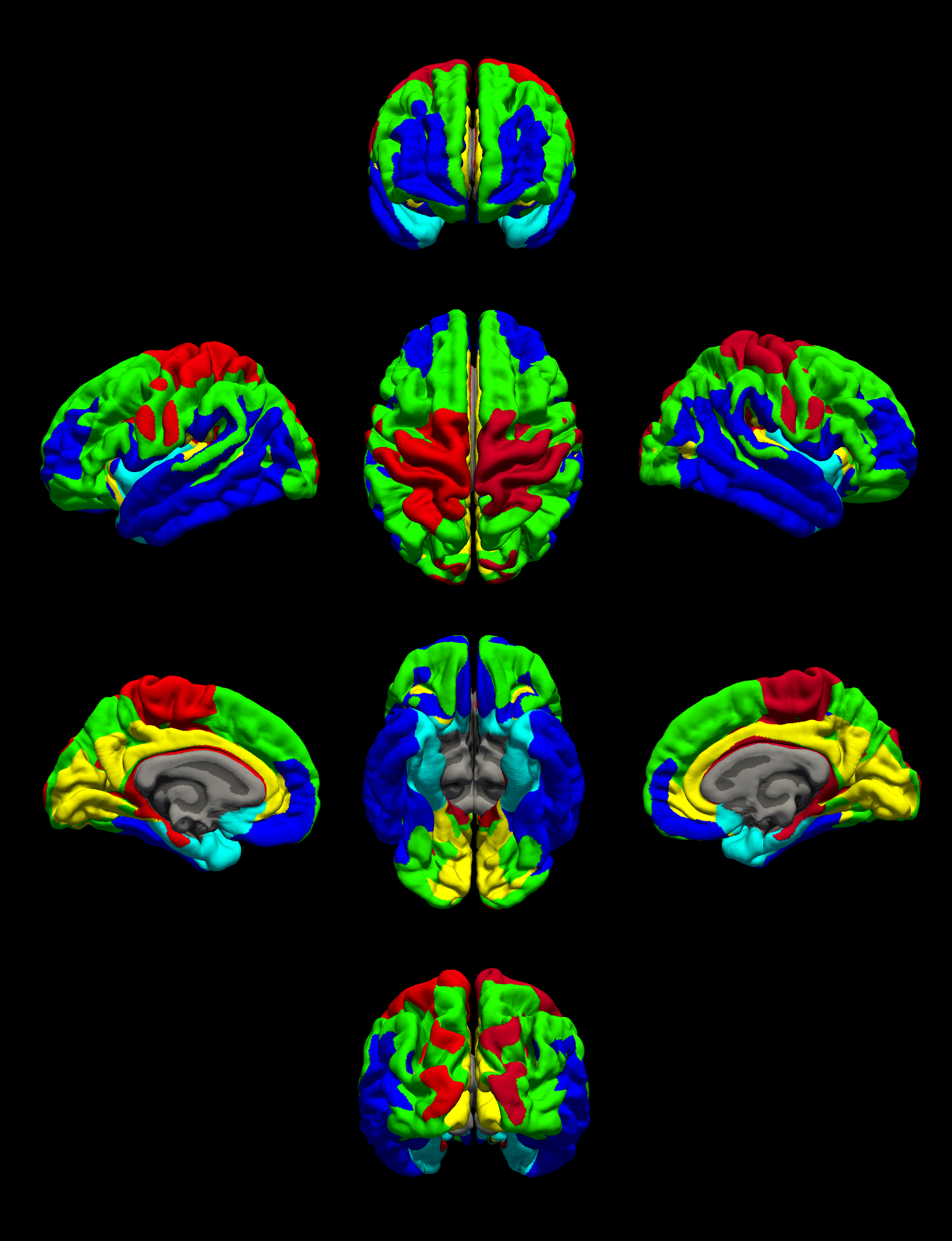

Supplement: Supplementary Data [file bhy249_supplementary_materials.zip › bhy249_Figure_2s.tif]
